# The Appearance and Behavior of the Pupil in Health and Disease

**Published:** 1888-12

**Authors:** T. J. Tyner

**Affiliations:** Austin, Texas


DANIEL'S

### Texas Medical Journal.

A Representative Organ of the Medical Profession, and an Exponent of Rational Medicine; devoted to the Organization, Advancement and Elevation of the Profession in Texas.

Published Monthly.—Subscription $2.00 a Year.

Vol. IV. AUSTIN, DECEMBER, 1888. No. 6.

### Original Articles.

CONTRIBUTED EXCLUSIVELY TO THIS JOURNAL.

*The Articles in this Department are accepted and published with the understanding that we are not responsible for, nor do we indorse the views and opinions of the writers by so doing.*

### THE APPEARANCE AND BEHAVIOR OF THE PUPIL IN HEALTH AND DISEASE.

By T. J. Tyner, M. D., Austin, Texas.

[Read before the Travis County Medical Society.]

IN THE treatment of this subject, I shall endeavor to show the value of pupillary phenomena, as a factor in the diagnosis of both local and general diseases.

It is scarcely necessary to add here, that a diagnosis of some remote disease, based upon the abnormal appearance of the pupil, without concomitant symptoms, and until all local causes which might give rise to abnormal changes have been excluded, would be unwarranted, and, as the significance of the pupil in general diseases would be of little or no value without a comprehensive knowledge of the changes which take place in local diseases, and even in health, a thorough study on this point will amply repay for the time so given.

The normal pupils should be of the same size and shape in each eye. To make a simple, but satisfactory examination, the patient should sit in front of a well-lighted window, and one eye shaded with the hand, or a dark cloth, so that all light is excluded. The pupil will now dilate, and on removing the shade, the patient at the same time opening the eye, it will be seen to rapidly contract to its former size. The other pupil will also act, though to a much less extent. The first is direct, the second indirect reflex action. The pupil contracts when the eye is fixed on a near object, say eight to twelve inches, which is owing to the effect of accommodation and convergence. If the eye is now directed to a distant object, the pupil dilates, though this is much less noticeable than in reflex action. In persons over fifty years of age, the pupil is contracted, and much less mobile than in those under that age, in whom the pupil is dilated. This is very clearly shown in the greater difficulty in making an ophthalmoscopic examination in old persons, and the ease with which it is done in the young. A deviation from this rule is generally pathological.

### LOCAL PATHOLOGY.

Unequal pupillæ are never seen in health, unless we except congenital anomalies, results of traumatism, and when there is a very great difference in the refraction of the two eyes.

The pupil may be immovable in iritic adhesions, and is generally contracted, and may be of irregular shape. This is very clearly shown by a few drops of a one per cent. solution of atropia in the eye, when the pupil will yield only at that portion of the iris not adherent, and the irregularities will be very striking. However, it sometimes happens that the entire pupillary edge of the iris is bound down, and absolutely immovable throughout, though in such a case the cause is so apparent it could not be identified with anything else. In atrophy of the iris from intra-ocular pressure in glaucoma, the pupil is dilated and immovable. The same is present in amaurosis due to disease of the retina, or optic nerve. When one eye only is blind, the pupil is less dilated, and may be, to a certain extent, mobile. This is, however, not due to reflex action, but associated action. Idiopathic mydriasis is almost always confined to one eye, and partial reaction to strong light may still be manifest, and the accommodation not wholly suspended, as only the sphincter may be paralyzed. It comes on suddenly, and as a rule passes off in the same manner, though it may last for a lifetime. In the mydriasis from atropia, duboisine, etc., both the accommodation and reflexes are absolutely suspended, and the pupil is dilated to its full extent, which is very rarely, if ever, the case in the other forms.

Idiopathic Myosis is very rare, and reaction very feeble. Even with strong solutions of atropia, the effect is slight. Why idiopathic mydriasis, is so much more frequently met with than this form of myosis, is probably due to the character of the nerve supply. The mechanism which causes the latter, is refex in its nature, and more susceptible to extraneous influences than the dilating, which is tonic in its action. However, this is a mere hypothesis, based only on clinical observation, and without the authority of any known physiological fact.

A word here as to the action of the nerves having dominion over the pupil will not be out of place. "The pupil is under the control of two antagonistic sets of nerves. One, the third, or rather branches of the third,—the efferent, the optic being afferent. This is the contracting mechanism, and refex in its nature; the other, a dilating mechanism, tonic in action, and of which the cervical sympathetlc is the efferent tract. Hence, any influence which inhabits one or the other, causes either dilatation or contraction. That is to say, if the third or optic nerve is divided, contraction does not only cease, but dilatation occurs at once, on account of the tonic dilating influence of the sympathetic being left without resistance, or antagonism. When the sympathetic is divided, contraction takes place." (Quoted from Foster, from memory).

The most frequent local disturbances producing contraction are, ciliary neuralgia, iritis, and all irritating diseases of the cornea. It may also be produced by long continued use of the eyes at minute objects, such as watch-making, engraving, etc., in consequence of which, the sphincter acquires a preponderating power over the dilator. Drugs, as eserine, pilocarpine, etc., cause rigid contraction, but the accommodation is only slightly affected, though refex action is lost for a time. The effect passes off in a few hours, while that of atropia continues for from six to eight days. This has always seemed to me an additional proof of the susceptibility of the reflex apparatus, or absence of a perfect antagonism, as we know of no reason why myotics should be more evanescent than mydriatics.

I come now to a part of the subject not so well understood, though no less inteiesting. The pupil in remote diseases.

In cerebral hyperæmia, congestion, excessive use of alcohol and opium, and the early stage of meningitis, the pupils are contracted. Paralysis of the dilating fibres indicates some alteration in the sympathetic, as in locomotor ataxy. In this affection the diameter is not affected by the strongest light, but continues to be manifest in accommodation and convergence. This is known as the Argyle-Robinson pupil, and is pathognomonic of locomotor ataxy.

In inflammation following injuiries of the brain, myosis is the rule. In concussion of the brain reflex action is lost, and regained very slowly, the length of .time depending on the extent of the injury, without either marked myosis or mydriasis.

Persistent, or frequently recurring unilateral dilatation sometimes precedes by several years, some mental aberration, or general paralysis, and should always be regarded as a symptom of great gravity, as it is in nearly every case, premonitory of central disturbance. In epileptic coma the pupils are dilated, and during the paroxysm, should they fail to react to light, any suspicion as to simulation would be out of the question. In hysteria and hysterical mania, the pupils are dilated; also in the paralytic form of rheumatism, or it may, in fact, does frequently occur, in the latter stages of syphilis; the poison setting up a periostitis in the spheniodal fissure ; or pressure of a syphilitic tumor, or finally, there may be syphilitic neuritis. Mydriasis frequently follows as a sequel to diphtheria. Injuries of the brain with a widely dilated pupil of one eye, indicates an intra-cranial hemorrhage on the same side, with a direct presure on the oculo-motor nerve in its course through the middle cranial cavity.

The ephemeral dilatation of the pupil at different periods of the day, is due to irritation of the sympathetic, and is a premonitory symptom of monomania. This is also present in helmenthiasis, the pupil rapidly dilating at varying intervals during the day.

In conclusion, I hope you will not think the following general remarks in bad taste :

The abnormal appearance of the pupil—particularly when unilateral—should never be disregarded, for it is frequently the first symptom of some of the gravest cerebral affections ; though for the most part, it is but one in an interminable group of symptoms, and can be only an aid in the diagnosis, except in rare cases.

To differentiate, and localize cerebral disturbances, is attended with more difficulties than any other undertaking in pathology, and can only be made with certainty, by a thorough knowledge of the anatomy of the brain, combined with much experience, which alone can enable us to correctly interpret the symptoms, as they are made manifest.

